# Plant‐eating carnivores: Multispecies analysis on factors influencing the frequency of plant occurrence in obligate carnivores

**DOI:** 10.1002/ece3.7885

**Published:** 2021-07-22

**Authors:** Hiroto Yoshimura, Satoshi Hirata, Kodzue Kinoshita

**Affiliations:** ^1^ Wildlife Research Center Kyoto University Kyoto Japan

**Keywords:** carnivore, diet, felids, plant eating

## Abstract

Plant‐eating behavior is one of the greatest mysteries in obligate carnivores. Despite unsuitable morphological and physiological traits for plant consumption, the presence of plants in scat or stomach contents has been reported in various carnivorous species. However, researchers’ interpretations of this subject are varied, and knowledge about it is scarce, without any multispecies studies. This study assessed the extent of variation in the frequency of plant occurrence in scat and stomach contents, as well as its relationship with various factors in 24 felid species using data from 213 published articles. Since the frequency of plant occurrence has not always been reported, we created two‐part models and estimated parameters in a Bayesian framework. We found a significant negative relationship between the frequency of plant occurrence and body mass. This may be because plant‐eating behavior reduces the energy loss caused by parasites and increases the efficiency of energy intake, which has a greater importance in smaller animals that have relatively high metabolic rates. This exploratory study highlights the importance of considering plant consumption in dietary studies on carnivorous species to understand the adaptive significance of this behavior and the relationship between obligate carnivores and plants.

## Graphical abstract

Plant‐eating behavior is one of the greatest mysteries in obligate carnivores. This study assessed the extent of variation in the frequency of plant occurrence in scat and stomach contents, as well as its relationship with various factors in 24 extant felid species using data from 213 published articles. We found a significant negative relationship between the frequency of plant occurrence and body mass.

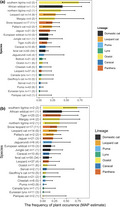

## 1 INTRODUCTION

The behavior, morphology, and physiology of animals are strongly influenced by their dietary traits, since adaptation for efficient feeding leads to increased fitness (Boag & Grant, 1981; Clauss et al., 2008; Grant & Grant, 2002; Janson & Boinski, 1992; Phillips & Shine, 2005; Pyke et al., 1977). Indeed, several species have various highly specialized traits incorporated in their diet (Grant & Grant, 2002; Janson & Boinski, 1992; Phillips & Shine, 2005; Smithsonian Institute, 2020). Hence, understanding their dietary needs is an effective means to study the behavior, ecology, and evolutionary history of target species.

Mammals can be roughly divided into three groups according to their diet: carnivores, omnivores, and herbivores. Plants are the primary food source of herbivores and are a relatively abundant resource in the environment. The composition of plant tissue is quite different from that of animals. Contrary to animal cell membranes, which mainly consist of proteins, plant cell walls are rich in carbohydrates, especially cellulose, which is difficult for animals to digest (Tomme et al., 1995; Watanabe & Tokuda, 2001). In addition, several plants contain toxic compounds as an antipredatory defense strategy (Dearing et al., 2005). Thus, herbivores have developed special digestive systems to detoxify secondary compounds and obtain nutrition from a plant‐based diet (Hofmann, 1989; Vallentine, 2000). In contrast, carnivores have predatory and scavenging feeding strategies, possessing numerous traits suitable for hunting and/or eating other animals. Feeding on other animals is nutritionally more efficient than eating plants, since the chemical composition of the food item is quite similar to that of the consumer (Hayami, 1967). Functional carnivores also have morphological and physiological trait characteristics of this diet. For instance, their dentition is better suited to slicing (Hamper et al., 2012; Van Valkenburgh, 1991), and their digestive tracts are shorter than those of herbivores (Stevens & Hume, 2004) owing to a decreased requirement for fermentation when digesting animal tissue as opposed to plant tissue. Additionally, taste receptor function is altered in many carnivores, including in felids; there is a loss of sensitivity to sugar in fruits and heightened sensitivity to amino acid and bitter compounds (Bosch et al., 2015; Jiang et al., 2012; Kim et al., 2016; Li & Zhang, 2014).

All members of Felidae are considered obligate carnivores, whose diets consist almost entirely of animal flesh, based on their dentition and physiological specialization (Legrand‐Defretin, 1994; Morris, 2002; Sanquist & Sanquist, 2002; Van Valkenburgh, 1991; Van Valkenburgh & Gittleman, 1989). These species are widely distributed and inhabit various environments, from the tropics to the frigid zones (Johnson, 2006; Kitchener et al., 2017). In several regions, these obligate carnivores [e.g., tigers (*Panthera tigris*) (Kapfer et al., 2011), snow leopards (*Panthera uncia*) (Shehzad et al., 2012)] eat plants even though their diet is considered to be exclusively carnivorous, and despite the aforementioned morphological and physiological traits that are not suitable for plant consumption. Yet, researchers’ interpretations of the presence of plant tissues in scat samples or stomach contents are varied, possibly owing to the difficulties associated with observing this plant‐eating behavior and because the amount of plant content present in these samples is often small. Some researchers believe that the presence of plant content is caused by unintentional intake (Avenant & Nel, 2002; De Villa Meza et al., 2002; Krofel et al., 2011), while others argue that there might be some advantages of plant eating (Hoppe‐Dominik, 1988; Sueda et al., 2008; Tatara & Doi, 1994; Xiong et al., 2016). Indeed, observational studies indicate that felids eat plants voluntarily (Montalvo et al., 2020; Yoshimura et al., 2020) both in the captivity and in the wild, which indicates that this behavior is relatively common and natural among felids. However, experimental studies suggest that cellulose intake can be disadvantageous, since it decreases dry matter and energy digestibility (Edwards et al., 2001; Prola et al., 2010). In addition, because of pseudogenization of the gene encoding a specific detoxification enzyme, felids are unable to detoxify phenolic compounds found in plants (Shrestha et al., 2011). Therefore, there may be some advantage for the existence of plant‐eating behavior in felids. Currently, three major hypotheses have been proposed to explain the adaptive significance of plant‐eating in carnivores. First is the self‐medication hypothesis (Hart, 2008). Many animals are known to use plants to counter parasites or diseases (Hart & Hart, 2018; Huffman, 2003; Huffman & Canon, 2000). Sueda et al. (2008) reported in a questionnaire survey of owners of dogs under one year of age that these dogs ate plants more frequently, and the authors suggested that plant consumption may be a way for individuals with low immunity to fight parasites and pathogens. Second is the hair evacuation hypothesis (Shultz, 2019; Yoshimura et al., 2020). Functional carnivores often ingest their own hair while grooming, as well as the hair of their prey. Ingested plants are considered to aid in excreting hairballs (Herbst & Mills, 2010). Third is the food source hypothesis. DNA extracted from leopard cat scats included *Solanum* and *Rosoideae* species that produce berry fruits rich in sugar and nutrients (Xiong et al., 2016). Although the replacement of animal food by fruits may be subject to physiological constraints (Larivière et al., 2001), fruits may help obligate carnivores endure starvation or periods when prey animals are scarce.

Currently, knowledge about the plant‐eating behavior of felids is scarce, and no comprehensive multispecies analyses have been performed. In this study, we attempted to explore and investigate factors that drive plant‐eating behavior of felids in order to understand the common features of this unique behavior among felid species. To clarify whether plant eating is conserved through the evolution of Felidae, we need to evaluate the relationship of this behavior with phylogenic history. Environmental factors also need to be considered since Felids are widely distributed throughout diverse habitats (Johnson, 2006; Kitchener et al., 2017). In addition, given that the body mass of animals affects their diet (Carbone et al., 1999; Kleiber, 1947), its effect should be examined as well. Therefore, in this study, we focused on the aforementioned factors to elucidate their relationship with the frequency of plant consumption in extant feline species.

## 2 MATERIALS AND METHODS

### 2.1 Literature search

A literature search using Web of Science (www.webofknowledge.com) was conducted on 15 September 2020 with the following keywords: “[common name of each species]” OR “[scientific name of each species]” AND “diet” OR “food.” Target species were all 41 extant felid species. Common names and scientific names were obtained from the International Union for Conservation of Nature (IUCN)/Species Survival Commission (SSC) cat specialist group (Kitchener et al., 2017). This search returned 4,100 research articles. The final output was based on the following exclusion criteria: review articles, captive studies (including domesticated animals), studies that were not based on scat or gut contents (e.g., an isotope study using body hair), and noncomprehensive studies (i.e., covered only specific food items). To assess the extent of variation in the frequency of plant occurrence in the diet of carnivores, we additionally sorted these studies according to the following exclusion criteria: sample size of less than 10 and species for which no studies calculated the frequency of plant occurrence. We separated data on fruits and other plants because fruits are different from other plants in terms of energy and nutrients. We only analyzed the data of nonfruit plants because the data of fruits were too scarce to be analyzed by itself. In all, 316 records from 213 studies of 24 felids (some references included records of several species) were used in the analyses (Appendix S1; Yoshimura et al., 2021).

### 2.2 Environmental factors

We included seven environmental attributes: absolute latitude, island size index, mean monthly precipitation, mean maximum daily temperature, mean minimum daily temperature, mean monthly normalized difference vegetation index (NDVI), and season (spring, summer, autumn, winter, dry, wet). In addition, we added sample type (scat or the digestive tract) because the remains present in the digestive tracts may be greater or lesser than those present in a single scat and may not be directly comparable. Latitude, precipitation, and temperature represent climate parameters of the habitat of subject animals. Since obligate carnivores live in diverse habitats, we added these factors to know whether frequency of plant occurrence relates to specific habitats. Animals on islands often show unique traits due to limited habitat and resources (Foster, 1964); therefore, we added “Island” as a binary variable, which reflects whether the sampling site was an island or mainland including a large island with area over 10,000 km^2^. We attempted to determine the effect of the abundance of vegetation on the frequency of plant occurrence in carnivores’ scat and stomach contents through NDVI. Season is mainly characterized by precipitation and temperature; thus, we used the mean values of the studied season for monthly precipitation and daily temperature to consider the seasonal difference. Where there was seasonal difference independent of precipitation or temperature, we added seasons as binary variables. Climate data were obtained from the MeteoBlue database (Cano‐Cruz & López‐Orozco, 2015). NDVI data from the Moderate Resolution Imaging Spectroradiometer (MODIS) onboard the Terra satellite were obtained using AppEEARS (AppEEARS Team, 2019). MODIS satellite was launched in 2000; therefore, we used the data from the oldest year available for the 89 records that started sampling before 2000. The variable mean monthly precipitation was normalized (scaled into a range of 0–1) to help the convergence of Markov chain Monte Carlo (MCMC) sampling. For further details about the collection of environmental data, see the Appendix S2 (Yoshimura et al., 2021).

### 2.3 Phylogenetic factors and body mass

Phylogeny of felids was based on Li et al. (2016). To test the phylogenetic signals in the mean frequency of plant occurrence in each species, phylogenetic eigenvector regression (PVR) was conducted (Diniz‐Filho et al., 1998). After extraction of pairwise phylogenetic distances from the branch duration information, the distance matrix was subjected to a principal coordinates (PCo) analysis. Following a broken‐stick model (Diniz‐Filho et al., 1998; Sakamoto et al., 2010), the first to fifth PCo axes (phylogenetic eigenvector 1–5, PV1‐5) were retained (Appendix S3: Table S1 and Figure S2; Yoshimura et al., 2021). These five axes cumulatively explained 86% of the total variance and were included in the analysis as predictor variables for measuring phylogenetic similarity. Additionally, log‐transformed body mass values were included as species‐specific factors. Body mass data of all species were according to Sakamoto et al. (2010). Since data concerning the body mass of the African wildcat (*Felis lybica*) were absent, we used the same value as that for the European wild cat (*Felis silvestris*), according to International Society for Endangered Cats Canada (International Society for Endangered Cats (ISEC) Canada, 2020).

### 2.4 Statistical analysis

All analyses were performed in R v.3.6.1 (R Development Core Team, 2019). To explain the number of samples that contained plant materials in each study, we constructed two‐part binomial (TPB) models. Since the frequency of plant occurrence has not always been reported in dietary studies on carnivores, several records in our dataset lacked values for frequency of plant occurrence. If we ignore records with missing values and apply ordinary regression models, it is likely to lead to imprecise estimation of parameters (Minami & Lennert‐Cody, 2013; Minami et al., 2007). Two‐part models are considered to be effective when dealing with data with many zero values or data generated from a combination of different mechanisms (Barry & Welsh, 2002; Matsuura, 2016a; Minami & Lennert‐Cody, 2013; Minami et al., 2007; Welsh et al., 1996). We assumed that the absence of reported plant material did not necessarily indicate that no plant material was found in the samples, as some reports mentioned that they ignored plant materials in scat or stomach samples (e.g., Abreu et al., 2008; Moleón & Gil‐Sánchez, 2003; Silva‐Pereira et al., 2011). Specifically, our models assumed that the frequency of plant occurrence has not always been reported irrespective of whether the samples included plant materials, and that the probability of reporting the frequency of plant occurrence follows a Bernoulli distribution with a parameter ψ. Thus,

$$\text{TPB}\left(y_{i},N_{i},\psi \right)=\text{Bernoulli}\left(0|\psi \right)\;\text{if}\;y_{i}=\text{NA,}$$

$$\text{TPB}\left(y_{i},N_{i},\psi \right)=\text{Bernoulli}\left(1|\psi \right)*\text{Binomial}(y_{i}|N_{i},p_{i})\;\text{if}\;y_{i}\neq \text{NA,}$$

where $y_{i}$ is the number of samples that contained plant materials, $N_{i}$ is the sample size, and $p_{i}$ is the frequency of plant occurrence in record *i*.

#### 2.4.1 Model 1: Variation in the frequency of plant occurrence in obligate carnivores

In this model, we assumed that the extent of intraspecies variation in the frequency of plant occurrence differs between species. Thus,

$$y_{i}∼\text{TPB}\left(\psi ,N_{i},p_{i}\right),$$

$$\text{logit}\left(p_{i}\right)=\alpha_{j}+\tau_{i},$$

$$\tau_{i}∼\text{Normal}\left(0,\theta_{j}^{2}\right),$$

where $\alpha_{j}$ represents the mean frequency of plant occurrence in species *j*, $\tau_{i}$ represents the random effect which explains the overdispersion between records, and $\theta_{j}$ is a hyperparameter vector with a length of the number of species (Appendix S2: Table A1).

#### 2.4.2 Model 2: Environmental and nonenvironmental factors affecting variation in the frequency of plant consumption in obligate carnivores

In this model, we explored the factors that affect the frequency of plant occurrence observed in each study. We assessed the effect of each variable using an approach similar to the hierarchical Bayesian models:

$$y_{i}∼\text{TPB}\left(\psi ,N_{i},p_{i}\right),$$

$$\text{logit}\left(p_{i}\right)=\alpha_{j}+\sum_{k=1}^{s}\beta_{k}*X\_\text{env}\left[i,k\right]+\tau_{i},$$

$$\alpha_{j}=I+\sum_{l=1}^{t}\varepsilon_{k}*X\_\text{sp}\left[j,l\right]+\varphi_{j},$$

$$\varphi_{j}∼\text{Normal}\left(0,\omega^{2}\right),$$

$$\tau_{i}∼\text{Normal}\left(0,\theta_{j}^{2}\right),$$

where $\alpha_{j}$ represents the species‐specific intercept of species *j*, β are coefficients of environmental factors *X_*env, *I* is the species‐independent intercept, ε are coefficients of nonenvironmental factors *X_*sp (i.e., body mass and phylogenetic eigenvectors), φ explains the overdispersion between species with hyperparameter ω, and τ explains the overdispersion between records with hyperparameter θ (Appendix S2: Table A1). The number of environmental and nonenvironmental factors is expressed as *s* and *t*, respectively. When considering the overdispersion between records, the standard deviation of τ was assumed to differ between species since different species had different distribution areas, number of references, etc. Thus, hyperparameter θ is a vector with a length corresponding to the number of species. To consider the effect of collinearity in Model 2, we examined the correlation between environmental factors and between nonenvironmental factors using Pearson's product–moment correlation (*r*), but |*r*| < 0.80 (Elith et al., 2006; Matsuura, 2016b) in all pairs.

### 2.5 Data imputation

We estimated parameters in the models mentioned above using the original dataset (Model 1_1 and Model 2_1). In these models, missing values in the frequency of plant occurrence are treated as the same NA. However, the presence of plant material in samples has been reported in some studies even if the frequency of plant occurrence has not been reported. These descriptions are informative since they mean that missing values were at least above zero. Therefore, we attempted to impute the missing data concerning the frequency of plant occurrence so that there was no waste of information. First, we sorted the literature without information regarding the frequency of plant occurrence into two groups: literature reporting the presence of plant materials in samples and those in which the presence of plant materials has not been reported. We then imputed and replaced the 23 records from 14 references in the first group using two different methods.

#### 2.5.1 Model 1_2 and Model 2_2: Data imputation with random values

First, random values were sampled from a sequence of 0.01 to 1 in increments of 0.01 to impute the frequency of plant occurrence. Then, the number of samples containing plant materials (*y*) was calculated as a product of random values and sample size *N* for each record that required imputation.

#### 2.5.2 Model 1_3 and Model 2_3: Data imputation from posterior distribution of models without data imputation

First, posterior distributions of parameter p in models without data imputation (Model 1_1 and Model 2_1) were transformed into frequency distributions. The minimum unit of bins was set as 0.005 in Model 1_3 and 0.01 in Model 2_3, respectively, to avoid the inclusion of all posterior distributions into the zero bins. Frequency distributions were then transformed into ratios to decide the sampling probability of each bin. Afterward, nonzero values were sampled according to this probability. Finally, the number of samples containing plant materials (*y*) was calculated as a product of p and sample size N of each dataset that required imputation. Since ψ represents the probability of the frequency of plant occurrence to be reported, estimation of ψ with the imputed dataset was considered to be inappropriate. Therefore, the parameter ψ was sampled from the posterior distribution of models without data imputation (Model 1_1 and Model 2_1).

### 2.6 Parameter estimation

We sampled all parameters using the No‐U‐Turn Sampler (Hoffman & Gelman, 2014) within an MCMC. We ran four parallel chains and calculated the potential scale reduction factor (Rhat; Gelman et al., 2013; Kruschke & Liddell, 2018) to check convergence. The number of iterations was set as 5,000 with 2,000 warm‐ups in the models without data imputation (Model 1_1 and Model 2_1). In models with data imputation (Model 1_2, Model 1_3, Model 2_2, and Model 2_3), MCMC sampling was repeated 10 times to reduce the potential effect of specific random value set. Thus, the number of each iteration was set as 2,000 with 1,500 warm‐ups to reduce computational load for these models, and posterior distributions from each trial were cumulated. This rate was 1/2, meaning that one of every two consecutive values of posteriors was taken to reduce autocorrelation. If Rhat was 1.0 or less, the model was considered successfully converged. In addition, we conducted graphical posterior predictive checks to determine whether our models were a good fit (Appendix S3: Figures S3 and S4; Yoshimura et al., 2021). Models coded in Stan were compiled into C++ and run using the “rstan” package (Carpenter et al., 2017). Weakly informative priors were used according to prior recommendations from the Stan development team (Prior, 2020) and “rstanarm” development team (Gabry & Prior, 2020). Specifically, intercepts ($\alpha_{j}$ and *I*) and coefficients (β and ε) follow Student's *t*‐distribution with three degrees of freedom [Student's *t* (3,0,5)] and hyperparameters followed an exponential distribution [exp(1)].

We used a mode of posterior distribution (maximum a posteriori, MAP) with an 89% highest density interval (HDI; Makowski et al., 2019) and a mean of posterior distribution (expected a posteriori, EAP) with a 95% Bayesian credible interval (CI) as the summary statistic. The MAP estimate is less susceptible to long tail of the posterior distribution. In contrast, the EAP estimate can indicate the tips of asymmetric posterior distribution. Thus, we reported both summary statistics. We used the HDI + ROPE (region of practical equivalence) decision rule as the basis for accepting or rejecting null values of fixed effects (Kruschke, 2018; Makowski et al., 2019). The “bayestestR” package (Makowski et al., 2019) was used to calculate MAP, HDI, and ROPE. According to Makowski et al. (2019), an 89% HDI is deemed to be more stable for an effective sample size less than 10,000. Estimated values were considered significant when the entire HDI fell outside the ROPE (i.e., the null hypothesis was rejected; (Kruschke, 2018; Makowski et al., 2019). The limits of the ROPE were set to the effect size at half of Cohen's conventional definition of a small effect (Cohen, 1998), that is, [−0.1, 0.1], proposed by Makowski et al. (2019) and Kruschke et al. (Kruschke, 2018; Kruschke & Liddell, 2018). The “rope” function was used to calculate the overlap of HDI and ROPE. Additionally, estimated values were considered significant when the 95% CI did not include zero (Kubo, 2018).

## 3 RESULTS

Within the 316 records that passed the exclusion criteria, the number of records dedicated to each species varied from 1 [African wildcat, Jungle cat (*Felis chaus*), Canada lynx (*Lynx canadensis*)] to 55 (feral cat).

Within the 316 records, the number of records that reported the frequency of plant occurrence was 118 (37%). As for the 198 records that did not calculate the frequency of plant occurrence, 23 mentioned plant materials and 175 did not mention plants at all.

We imputed missing data with description about the presence of plants using two methods when estimating parameters. The methods used to estimate parameters when imputing missing data concerning the frequency of plant occurrence did not affect the conclusion of the analysis. Therefore, we mainly used the results obtained from data‐imputed models (Model 1_3 and Model 2_3). Figures and tables for the other models are included in the Appendix S3: Tables S2–S4 and Figures S5–S7 (Yoshimura et al., 2021).

**FIGURE 1:**
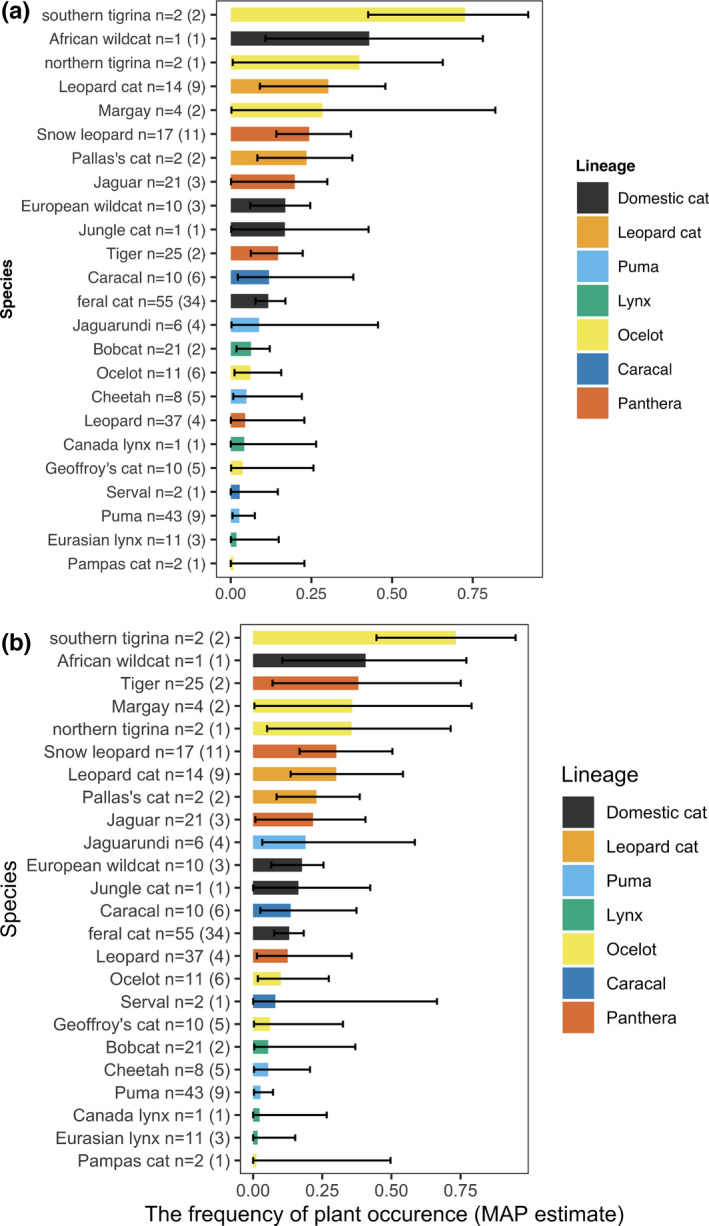
Estimated frequency of plant occurrence (a) maximum a posteriori estimate with the 89% highest density interval (HDI), (b) expected a posteriori estimate with the 95% credible interval (CI) of each species using Model 1_3. The numbers next to the common names of species represent the numbers of records, and the numbers in the parentheses are the numbers of records showing the frequency of plant occurrence values

The frequency of plant occurrence varied substantially, from 0.005 [Pampas cat (*Leopardus colocola*)] to 0.749 (southern tigrina (*Leopardus guttulus*); Table 1, Figure 1, Appendix S3: Figures S5–S7; Yoshimura et al., 2021).

**TABLE 1:** Estimated frequency of plant occurrence in carnivores in Model 1_3

Lineage	Common name	Academic name	Number of records	MAP estimate [lower HDI, upper HDI]	EAP estimate [lower CI, upper CI]
Domestic cat	Feral cat	*Felis catus*	55 (34)	0.122 [0.077, 0.167]	0.124 [0.073, 0.187]
	Jungle cat	*Felis chaus*	1 (1)	0.162 [0, 0.443]	0.237 [0.018, 0.793]
	African wildcat	*Felis lybica*	1 (1)	0.396 [0.087, 0.756]	0.436 [0.057, 0.903]
	European wildcat	*Felis silvestris*	10 (3)	0.172 [0.063, 0.248]	0.167 [0.053, 0.317]
Leopard cat	Pallas's cat	*Otocolobus manul*	2 (2)	0.238 [0.084, 0.389]	0.247 [0.071, 0.534]
	Leopard cat	*Prionailurus bengalensis*	14 (9)	0.298 [0.119, 0.507]	0.327 [0.12, 0.608]
Puma	Cheetah	*Acinonyx jubatus*	8 (5)	0.056 [0.004, 0.213]	0.11 [0.014, 0.366]
	Jaguarundi	*Herpailurus yagouaroundi*	6 (4)	0.078 [0.003, 0.494]	0.248 [0.022, 0.725]
	Puma	*Puma concolor*	43 (9)	0.027 [0.004, 0.073]	0.042 [0.007, 0.114]
Lynx	Canada lynx	*Lynx canadensis*	1 (1)	0.029 [0, 0.264]	0.13 [0.006, 0.644]
	Eurasian lynx	*Lynx lynx*	11 (3)	0.022 [0, 0.147]	0.073 [0.003, 0.361]
	Bobcat	*Lynx rufus*	21 (2)	0.062 [0.017, 0.128]	0.093 [0.025, 0.456]
Ocelot	Pampas cat	*Leopardus colocola*	2 (1)	0.005 [0, 0.212]	0.089 [0.001, 0.438]
	Geoffroy's cat	*Leopardus geoffroyi*	10 (5)	0.033 [0, 0.227]	0.112 [0.008, 0.404]
	Southern tigrina	*Leopardus guttulus*	2 (2)	0.749 [0.444, 0.94]	0.673 [0.23, 0.924]
	Ocelot	*Leopardus pardalis*	11 (6)	0.074 [0.02, 0.177]	0.105 [0.025, 0.26]
	Northern tigrina	*Leopardus tigrinus*	2 (1)	0.403 [0.226, 0.684]	0.44 [0.174, 0.81]
	Margay	*Leopardus wiedii*	4 (2)	0.416 [0.01, 0.807]	0.456 [0.03, 0.926]
Caracal	Caracal	*Caracal caracal*	10 (6)	0.127 [0.031, 0.396]	0.213 [0.038, 0.559]
	Serval	*Leptailurus serval*	2 (1)	0.024 [0, 0.129]	0.079 [0.007, 0.612]
Panthera	Jaguar	*Panthera onca*	21 (3)	0.144 [0.008, 0.328]	0.195 [0.026, 0.537]
	Leopard	*Panthera pardus*	37 (4)	0.051 [0.001, 0.23]	0.115 [0.012, 0.391]
	Tiger	*Panthera tigris*	25 (2)	0.132 [0.091, 0.207]	0.147 [0.079, 0.234]
	Snow leopard	*Panthera uncia*	17 (11)	0.259 [0.15, 0.392]	0.274 [0.141, 0.449]

Note

The numbers in parentheses represent the number of records that calculated the frequency of plant occurrence values. Estimated frequency is shown as maximum a posteriori (MAP) estimate (the mode of posterior distribution) with 89% highest density interval and expected a posteriori (EAP) estimate (the mean of posterior distribution) with 95% credible interval.

**FIGURE 2:**
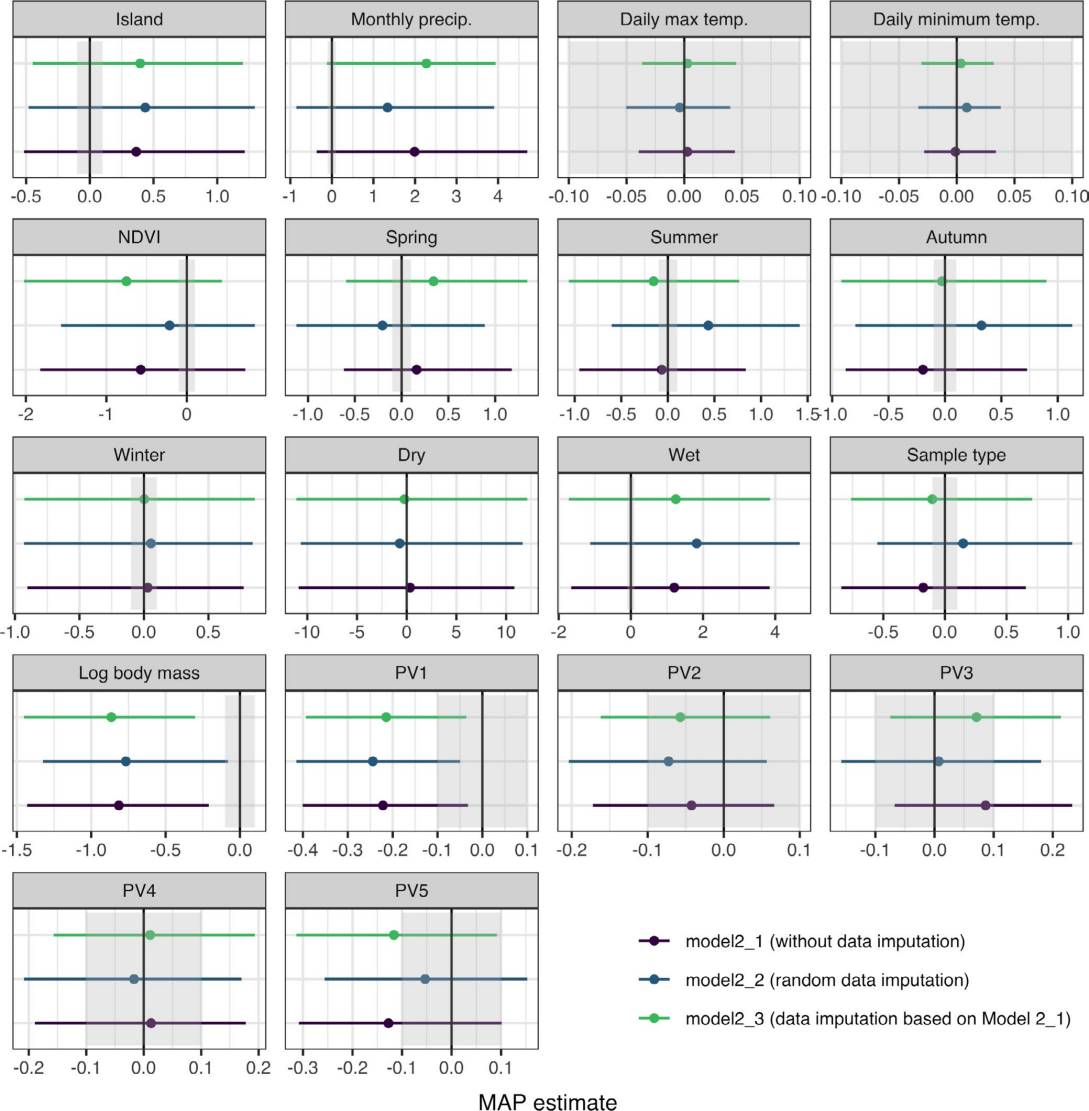
Maximum a posteriori (MAP; the mode of the posterior distribution) estimates of coefficients of fixed effects. The error bars represent 89% highest density interval, and the gray area represents the region of practical equivalence (ROPE). The black line indicates zero. Estimated parameters were considered as significant if the 89% HDI falls off from the ROPE

**FIGURE 3:**
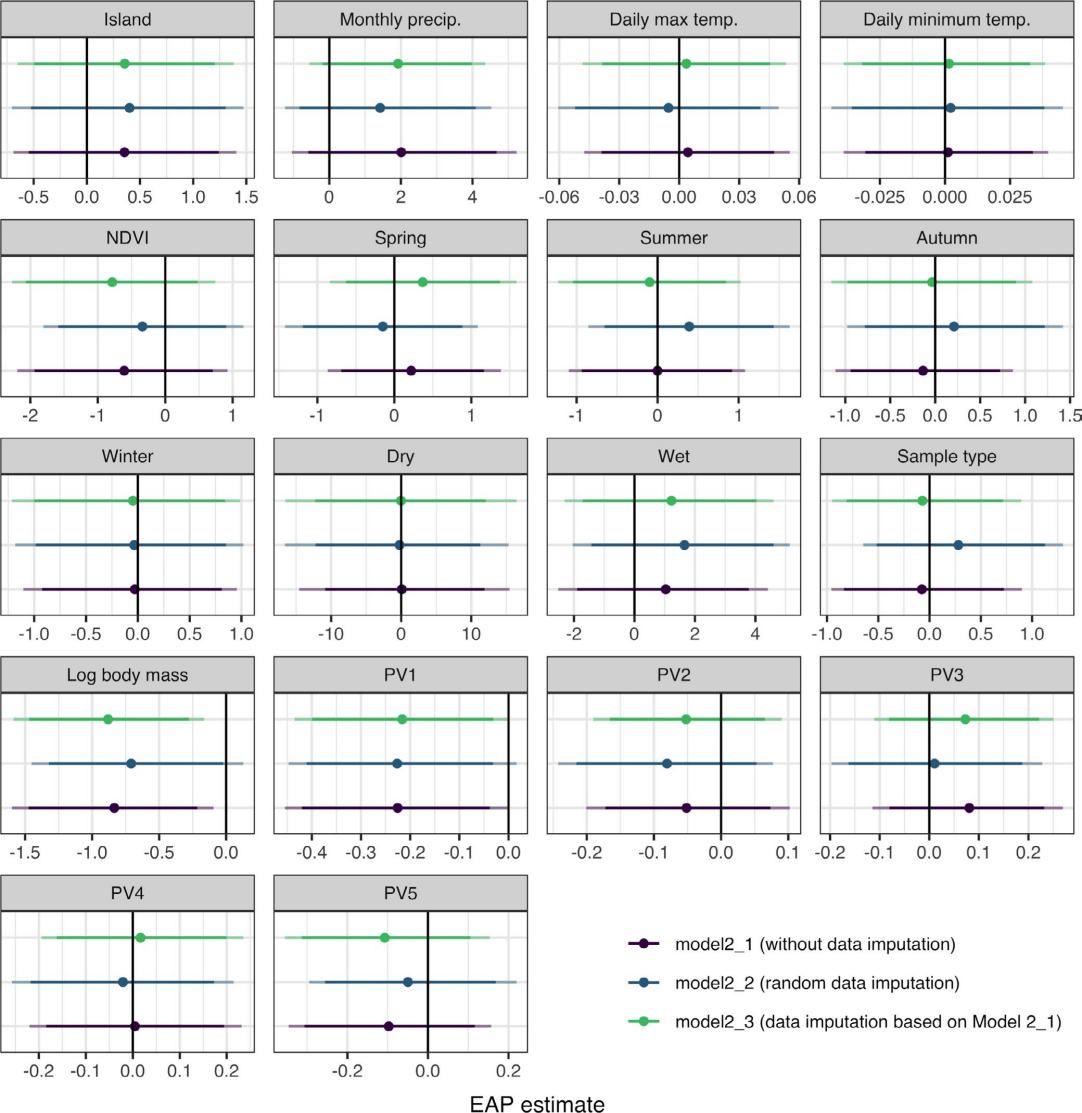
Expected a posteriori (EAP; the mean of the posterior distribution) estimates of coefficients of fixed effects. The light and thick error bars represent 95% and 90% credible interval (CI), respectively. The black line indicates zero. Estimated parameters were considered as significant if the 95% CI did not include zero

**FIGURE 4:**
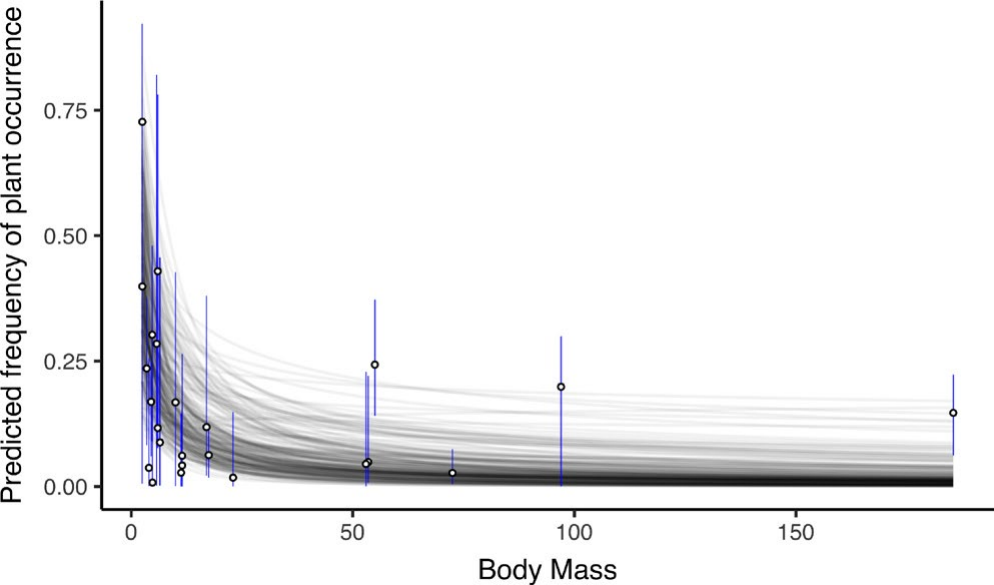
Response curve of the frequency of plant occurrence to body mass in Model 2_3. Dots are maximum a posteriori estimates of the frequency of plant occurrence, and error bars represent the 89% highest density intervals. Intercepts and slopes were randomly selected from posterior distributions

Within the 18 variables considered in Model 2, log‐transformed body mass (MAP = −0.814 [−1.452, −0.302], EAP = −0.881 [−1.586, −0.164]) had a significant effect on the frequency of plant occurrence based on the HDI + ROPE rule (Figures 2, 3, 4, Table 2). In addition, “PV1” (MAP = −0.222 [−0.393, −0.036], EAP = −0.216 [−0.435, −0.0002]) was also considered significant, since the 95% CI did not include zero (Figure 3, Table 2).

**TABLE 2:** Estimated coefficients of fixed effects in Model 2_3

Fixed effects	MAP estimate [lower HDI, upper HDI]	%HDI inside the ROPE	EAP estimate [lower 95% CI, upper 95% CI]	EAP estimate [lower 90% CI, upper 90% CI]
Island	0.364 [−0.448, 1.203]	0.13	0.356 [−0.651, 1.382]	0.356 [−0.495, 1.204]
Monthly precip.	1.991 [−0.119, 3.938]	0.024	1.919 [−0.553, 4.355]	1.919 [−0.195, 3.982]
Mean daily max temp.	0.003 [−0.037, 0.045]	1	0.004 [−0.048, 0.053]	0.004 [−0.039, 0.045]
Mean daily minimum temp.	−0.001 [−0.031, 0.032]	1	0.002 [−0.039, 0.038]	0.002 [−0.032, 0.033]
NDVI	−0.573 [−2.024, 0.439]	0.069	−0.785 [−2.273, 0.744]	−0.785 [−2.068, 0.48]
Spring	0.161 [−0.595, 1.343]	0.115	0.369 [−0.838, 1.588]	0.369 [−0.629, 1.374]
Summer	−0.066 [−1.063, 0.766]	0.141	−0.099 [−1.228, 1.025]	−0.099 [−1.046, 0.845]
Autumn	−0.195 [−0.919, 0.9]	0.156	−0.034 [−1.155, 1.08]	−0.034 [−0.975, 0.9]
Winter	0.029 [−0.927, 0.86]	0.152	−0.047 [−1.215, 0.987]	−0.047 [−0.999, 0.842]
Dry	0.337 [−11.104, 12.13]	0.016	−0.031 [−16.56, 16.47]	−0.031 [−12.282, 12.067]
Wet	1.202 [−1.72, 3.855]	0.038	1.224 [−2.306, 4.603]	1.224 [−1.712, 4.036]
Sample type	−0.176 [−0.762, 0.708]	0.179	−0.07 [−0.951, 0.894]	−0.07 [−0.807, 0.715]
Log body mass	**−0.814 [−1.452, −0.302]**	**0**	**−0.881 [−1.586, −0.164]**	**−0.881 [−1.469, −0.278]**
PV1	−0.222 [−0.393, −0.036]	0.135	**−0.216 [−0.435, −0.0002]**	−**0.216 [−0.399, −0.031]**
PV2	−0.042 [−0.162, 0.061]	0.771	−0.052 [−0.19, 0.09]	−0.052 [−0.165, 0.065]
PV3	0.086 [−0.075, 0.214]	0.611	0.073 [−0.111, 0.251]	0.073 [−0.081, 0.221]
PV4	0.013 [−0.157, 0.193]	0.669	0.017 [−0.194, 0.235]	0.017 [−0.162, 0.199]
PV5	−0.127 [−0.313, 0.092]	0.436	−0.107 [−0.355, 0.154]	−0.107 [−0.313, 0.106]

Note

Estimated frequency is shown as maximum a posteriori (MAP) estimate (the mode of posterior distribution) with 89% highest density interval (HDI) and expected a posteriori (EAP) estimate (the mean of posterior distribution) with 95% and 90% credible intervals (CI). Bold characters represent significant fixed effects. Estimated parameters were considered as significant if the 89% HDI falls outside the region of practical equivalence (ROPE) [−0.1, 0.1] or 95% CI did not include zero.

**FIGURE 5:**
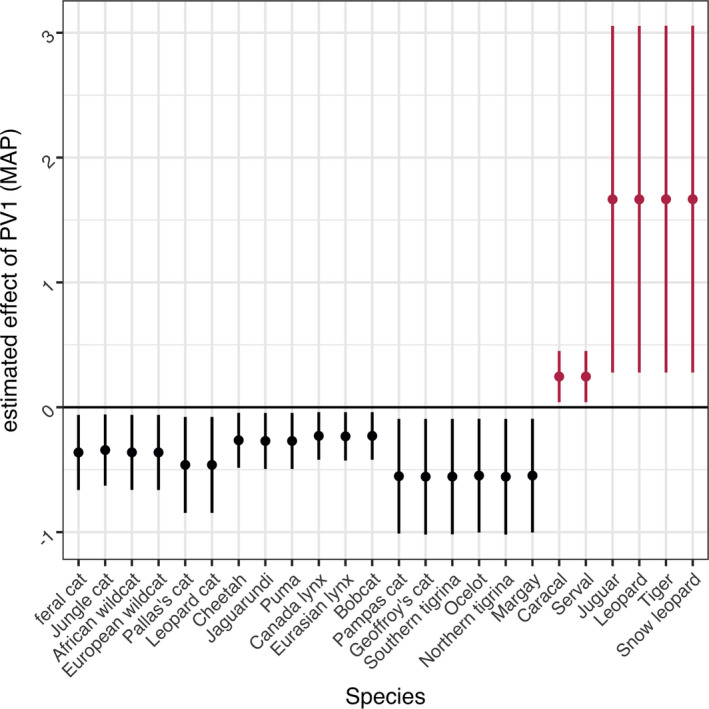
The products of PV1 and maximum a posteriori estimates for each species in Model 2_3. Error bars reflect the 89% highest density interval of each coefficient. Positive values are represented in red and negative values in black

Regarding the PVR (Diniz‐Filho et al., 1998; Sakamoto et al., 2010), PV1 tended to have a significant positive effect on the frequency of plant occurrence in the *Panthera* and *Caracal* genera and a negative effect in other felid lineages. This effect was greater in *Panthera* than in *Caracal* (Figure 5). Greater body mass was associated with a reduction in the frequency of plant occurrence with a probability of 95% when estimated in a one‐variable model, although the 95% CI included zero. However, PV1 showed a positive correlation with the frequency of plant occurrence with a probability of only 40%.

## 4 DISCUSSION

### 4.1 Limitations

Our data relied on the frequency of occurrence data from previous studies. Therefore, we should acknowledge the biases and limitations of the frequency of occurrence method (reviewed in Klare et al. (2011)). The frequency of occurrence method tends to overestimate the importance of small food items as it weighs the presence of small and large food items in the scats equally (Klare et al., 2011; Weaver, 1993). Although the frequency of occurrence is not always equivalent to the composition of the diet, Klare et al. (2011) stated that the frequency of occurrence per scat can contribute useful information about rare food items and help us understand a carnivore's ecology. In the present study, we did not evaluate the importance of plants relative to other items for felids nor did we seek to argue that plants make up most of their diet. Rather, we attempted to estimate the frequency of plant consumption by felids and identify the factors that could affect it. Although the frequency of plant occurrence per scat/gut sample can provide valuable information on how often wild cats consume plants, further accumulation of knowledge using biomass calculation helps achieve a more precise assessment (Klare et al., 2011) of the importance of plant consumption in carnivores.

The present study investigated the effect of environmental attributes, which represent the traits of research areas. We could not find a clear relationship between environmental factors and the frequency of plant occurrence. However, it should be noted that it is likely that more detailed factors, such as abundance of specific plant taxa or risk of parasite infection, that were not analyzed in the present study have correlations with the frequency of plant occurrence in felids. As the plant occurrence data were based on indirect evidence (scat, remains of the digestive content), it was difficult to obtain fine‐scale spatial and temporal environment data from the habitats of subject animals. This might have masked the effect of environmental factors. For example, we used mean of NDVI during the sampling period, but it was possible that a drastic vegetation change occurred during the sampling period or during the period we did not have NDVI data for. Although seasonal difference was considered in our models, several studies have reported the frequency of plant occurrence as data throughout the year, which possibly masked the actual seasonal patterns.

Hoppe‐Dominik (1988) suggested that leopards may eat plants to relieve hunger during periods of starvation. It is possible that physiological condition could confound with environmental factors. Further individual‐based studies are required to test the effect of physiological conditions.

### 4.2 Phylogenetic factors

The results showed that the frequency of plant occurrence was observed to be higher in *Panthera* and *Caracal*, the two earliest diverging lineages of Felidae (Kitchener et al., 2017; Li et al., 2016) than other felids. This might indicate that plant‐eating behavior in felids is a trace of omnivorous ancestral traits (Bradshaw, 2006; Tseng & Flynn, 2015a,b). However, this effect was not significant in the HDI + ROPE rule. Besides, *Panthera* consists entirely of big cats, thus the positive effect of PV1 on the frequency of plant occurrence in *Panthera* species conflicted with the negative effect that body mass was found to have on this variable. Hence, we confirmed the effect of both variables through one‐variable models and found that PV1 itself was not correlated with the frequency of plant occurrence. The significant effect of PV1 in the *Panthera* lineage may have been caused by the high frequency of plant occurrence relative to the body mass of these big cats. Although the result did not exclude the possibility that phylogeny shows a relationship with the frequency of plant occurrence in felids, it was likely to have little effect.

### 4.3 Body mass

We found that body mass showed a significant negative correlation with the frequency of plant occurrence, meaning that smaller carnivore species engaged in plant‐eating behavior more frequently than larger species. The correlation was significant in Model 2_1 and Model 2_3 according to both the HDI + ROPE rule and 95% CI, but not in the model with random data imputation (Model 2_2). However, the percentage of posteriors in the ROPE was only 3.9%, and 90% CI did not include zero in Model 2_2 (Figure 3, Appendix S3: Table S4; Yoshimura et al., 2021). In this model, the frequency of plant occurrence was imputed completely at random; therefore, unrealistic values such as 1 might have been applied and affected the posterior distribution. Hence, judging from the overall results, we concluded that body mass has a significant negative correlation with the frequency of occurrence.

One possible explanation for this correlation relates to self‐medication. Kleiber's law states that relative energy consumption is higher in smaller species (Kleiber, 1947). Maintenance metabolism (i.e., the energy required to maintain homeostasis) scales fractionally with body size; as such, smaller animals require more metabolic energy per unit of body mass (Demment & Van, 1985). Therefore, energy loss caused by parasites has higher consequences for smaller carnivores. Moreover, Gregory et al. (Gregory et al., 1996) suggested that host species with higher metabolic rates for their body size may show a greater number of parasite species due to increased food intake. A multispecies study of mammals in Mexico revealed that the order Carnivora showed the greatest occurrence of parasitic helminths and that the host body mass has significant negative correlation with parasite richness (Villalobos‐Segura et al., 2020). These studies support that the cost of parasites is higher in smaller felids than larger species. However, the association between parasite species richness and body weight varies depending on the subject species (Dáttilo et al., 2020); hence, further quantitative study is required to confirm the relationship between host body mass and parasite richness in felids. Several animal species are known to utilize plant physical or chemical aspects against parasites or pathogens (Bosch et al., 2015; de Roode et al., 2013; Hart & Hart, 2018; Huffman, 2003). Consumption of grasses is considered to work as scouring agent against intestinal parasites such as roundworms and tapeworms in canids (Bosch et al., 2015). Small carnivores might eat plants for parasite control, since the energetic costs of parasite load are relatively high. Leopard cat (*Prionailurus bengalensis*) scat has been reported to contain parasites on *Arundinella hirta* plant (Lee et al., 2014). Nonetheless, to our knowledge, this is the only study reporting the presence of plant and parasite in the same scat of felids.

Evacuation of hair or undigested materials can be another explanation. Plant‐eating behavior in felids is hypothesized to have an effect on hairball evacuation (Herbst & Mills, 2010; Shultz, 2019). Similar to the aforementioned endoparasites, a greater frequency of plant occurrence in small felids may relate to the high energy cost of an ectoparasite load. Fleas are the main ectoparasite that affect cats, and self‐grooming using cornified papillae on the tongue is one of the removal strategies (Hart & Hart, 2018). As the cost of ectoparasite load increases, the intensity of grooming increases, which is likely to result in increased ingestion of its own hair by the animal.

Carnivores weighing less than 21.5 kg generally consume animals consisting of 45% or less of their own mass, while those weighing more than 21.5 kg prey mostly on animals larger than themselves (Carbone & Gittleman, 2002). Small prey consumption often includes the ingestion of indigestible parts such as fur, skin, bone, and connective tissue, besides muscle and organs, while large carnivores can selectively eat digestible parts (Clauss et al., 2010; Stirling & McEwan, 1975). In humans, dietary fiber intake is known to promote digestion and bowel movements by stimulating peristalsis and mucus secretion in the digestive tract (Chutkan et al., 2012; El‐Salhy et al., 2017). Plant consumption might promote digestion or excretion of indigestible food items, which are consumed by small carnivores at a high frequency. Sugar cane‐derived fibers reduced the size of hairballs in the scat of domestic cats (Loureiro et al., 2014). However, cellulose, one of the main insoluble fibers, did not have such an effect (Loureiro et al., 2014), and plant intake had little effect on hair evacuation in captive snow leopards (Yoshimura et al., 2020). Owing to the aforementioned attributes of prey items, smaller carnivores are considered to be more tolerant to indigestible food items (Jethva & Jhala, 2004; Rühe et al., 2008). Indeed, Vester et al. (2008) demonstrated that small felids have higher digestion ability of dietary fiber, and Kerr et al. (2013) showed that tract dry matter, organic matter, fat, and energy digestibility coefficients decreased linearly with body weight in four medium‐to‐large cats [jaguar (*Panthera onca*), cheetah (*Acinonyx jubatus*), Malayan tiger (*Panthera tigris corbetti*), and Siberian tiger (*Panthera tigris altaica*)] fed cellulose and beet pulp diets. Although cellulose intake reduces dry matter and energy digestibility both in large (Kerr et al., 2013) and small felids (Edwards et al., 2001; Prola et al., 2010), smaller animals may be less affected, which could explain their increased tolerance to more frequent plant consumption. Nevertheless, this can be true whether or not plant intake has some adaptive significance for obligate carnivores, and thus, this does not negate the self‐medication hypothesis or the hair evacuation hypothesis.

## 5 CONCLUSION

This study summarized the current knowledge about plant‐eating behavior in carnivores and investigated its relationship with various factors. To date, little attention has been paid to the presence of plants in dietary studies of carnivores. Lack of information about plant eating in a report does not necessarily mean that plant occurrence in samples was absent in that study. Therefore, if we had only used the data of studies that report the frequency of plant occurrence values, the analyses would have been biased. To avoid this, our methods made the best use of all information available using two‐part models and Bayesian framework. We demonstrated the negative relationship of the frequency of plant occurrence with body mass. As the present study is exploratory, we cannot completely deny the alternatives. Nonetheless, our findings indicate that plant eating may have some functional significance as functional behaviors have a greater importance for smaller species that need to increase the efficiency of nutrient intake. Increased efficiency is achieved by not only increasing nutrient intake but also preventing the decrease in nutrient intake (e.g., parasites). Smaller species did not always present a higher frequency of plant occurrence than that did larger species; this may be owing to the various reasons for plant consumption and the fact that the frequency of intake varied with the primary role of the plant material. Further research is required to understand the evolution and adaptive significance of plant eating in carnivores. In particular, studies identifying plant species and their frequency of occurrence in wild carnivore samples using recently developed molecular biological methods (Monterroso et al., 2019) are important to infer the role of plant intake. Hypothesis‐centered studies will provide direct evidence about the adaptive significance of plant eating as well. By unraveling the relationship between carnivores and plants, we will be able to understand not only their behavioral ecology but also their interactions within ecosystems.

## CONFLICT OF INTERESTS

We have no competing interests.

## AUTHOR CONTRIBUTION

**Hiroto Yoshimura: Funding acquisition (lead);** Conceptualization (lead); Data curation (lead); Formal analysis (lead); Investigation (lead); Methodology (lead); Visualization (lead); Writing‐original draft (lead). **Satoshi Hirata:** Supervision (equal); Writing‐review & editing (equal). **Koduze Kinoshita:** Supervision (equal); Writing‐review & editing (equal).

## Data Availability

Data, codes, and Appendices S1‐S3 can be accessed from the Dryad Digital Repository: https://doi.org/10.5061/dryad.x95x69phj (Yoshimura et al., 2021).

## APPENDIX 1

Here, we describe the characteristics of plant eating in each lineage. According to Li et al. (2016), there are eight extant felid lineages. However, only seven lineages are described here because we did not have the data from Bay cat lineage.

### Domestic cat lineage

This represents the most recent lineage and consists of smaller species (Li et al., 2016). Among the 67 records found for this group, 55 described feral cats. There were 6 studies reporting fruit detection (Biró et al., 1999; Carvalho & Gomes, 2004; Ferreira et al., 2014; Lanszki et al., 2016; Meckstroth et al., 2007; Spencer et al., 2014), with fruit possibly having been consumed as food. However, fruit was detected more frequently in domesticated cats than in feral cats living on a Croatian island (Lanszki et al., 2016), suggesting that the detection of fruit content might be associated with proximity to human activity [e.g., food provisioning or scavenging garbage (Yamane et al., 1994)]. Additionally, there were several studies showing the presence of nonfruit‐bearing plants, which may have other benefits, such as parasite control (Hart, 2008; Hart & Hart, 2018; Sueda et al., 2008).

### Leopard cat lineage

This group consists mainly of small species inhabiting Central to South‐East Asia. The two species used in this analysis had a relatively high frequency of plant occurrence. Parasites, together with *A. hirta*, were detected in leopard cat scat in Korea (Lee et al., 2014), implying that plants likely contributed to antiparasite measures or promoted gastrointestinal tract movement (Tatara & Doi, 1994). Although no cases of fruit detection have been reported in the leopard cat, a DNA‐based study of its scat contents in China showed fruit‐bearing species, suggesting its use as food (Xiong et al., 2016).

### Puma lineage

This group consisted of three species, each belonging to a different genus and differing in both body size and distribution range. Overall, the frequency of plant occurrence in this group was low, although one study showed a high frequency of plant occurrence in the scat of the smallest species, the jaguarundi (*Herpailurus yagouaroundi*) (Kasper et al., 2016). This high degree of intraspecies variation is reflected as a wide range of HDI [0.003, 0.494] and CIs [0.022, 0.725] (Table 1). With the exception of one study on cheetahs (*Acinonyx jubatus*) in Iran (Zamani et al., 2017), no fruit was detected. Samples of Jaguarundi and pumas have been reported to contain 28% and 20% of Cyperaceae plants, respectively (Rocha‐Mendes et al., 2010). The higher presence of this family may be because of the distinctive surface of most of these grasses, which makes them easier to identify by texture, and because they tend to contain fewer toxic compounds (Hoppe‐Dominik, 1988).

### Lynx lineage

This lineage included the relatively large Eurasian lynx (*Lynx lynx*) and three medium‐sized species that are widely distributed in the Northern Hemisphere. The frequency of plant occurrence was relatively low in this group. Nevertheless, 7 studies reported the presence of fruit, and Mckinney and Smith (2007) reported that bobcats (*Lynx rufus*) in the Sonoran Desert fed more frequently on fruits and seeds during winter and spring droughts than on reptiles. Therefore, in this lineage, plants may serve mainly as a supplementary food source.

### Ocelot lineage

This group consisted of small species from Central to South America; six of the eight extant species (Kitchener et al., 2017) were used in this analysis. Fruit consumption has not been reported. The frequency of plant occurrence was high for three species and low for the others. Although these species have broad habitat selectivity, southern tigrina (*Leopardus guttulus*), northern tigrina (*Leopardus tigrinus*), margay (*Leopardus wiedii*), and ocelot (*Leopardus pardalis*) are more likely to inhabit less dry habitats (IUCN/SSC Cat Specialist Group, 2018). Indeed, the mean monthly precipitation was higher in the habitats of these four species (105, 106, 79, and 111 mm, respectively) than in the habitats of other species (Geoffroy's cat (*Leopardus geoffroyi*): 38 mm; Pampas cat (*Leopardus colocola*): 34 mm). Hence, the high frequency of plant occurrence in the three species may reflect hot and humid habitats where the risk of parasite and pathogen infection is relatively high (Froeschke et al., 2010; Kołodziej‐Sobocińska, 2019). The ocelot's larger body mass might have caused its relatively low frequency of plant occurrence compared with that for smaller species. Additionally, ocelots, pumas, and jaguars (*Panthera onca*) have been observed eating wild rice containing high levels of cyclooxygenase inhibitors (Montalvo et al., 2020), which works as an anti‐inflammatory agent in dogs and cats (Jones & Budsberg, 2000). However, it should be noted that studies on this topic are scarce and there is a high degree of uncertainty in the estimates.

### Caracal lineage

This lineage consists of medium‐sized species that live mainly in Africa. Caracals (*Caracal caracal*) had a higher frequency of plant consumption than servals (*Leptailurus serval*). The presence of nonfruiting plants has often been reported, and there was a study of caracals feeding on tsama melons (Melville et al., 2004). Melville et al. (2004) also found Kalahari sour grass (*Schmidtia kalihariensis*) in 38.8% of caracal scat. This is the dominant species in the Kalahari Desert, which only grows for a short period after sufficient rainfall (Dippenaar‐Schoeman et al., 2018), has a distinctive odor, and has glands that secrete acidic substances (Dippenaar‐Schoeman et al., 2018). Caracals may eat this plant to ingest these compounds possibly for self‐medication (Hart & Hart, 2018; Huffman, 2003) or for pH control in the digestive tract (Kerr et al., 2013), although it is unclear whether these compounds have a beneficial effect. This finding further suggests that these animals might use plant odor as one of the selecting factors for consumption.

### Panthera lineage

These so called “big cats” constitute one of the basal lineages of extant felid species (Li et al., 2016). Fruit consumption has not been reported for them; however, the presence of grasses and shrubs has been detected in numerous cases (e.g., Jumabay‐Uulu et al., 2014; Ott et al., 2007; Tkachenko, 2012). Hoppe‐Dominik stated that leopards (*Panthera pardus*) may eat grasses to keep their digestive tract moving during starvation (Hoppe‐Dominik, 1988). However, captive snow leopards also ate plants regularly even though they were fed daily (Yoshimura et al., 2020), suggesting that starvation is not always the trigger for plant eating. Furthermore, it has been suggested that grasses are selectively eaten because they are free of secondary plant compounds, unlike those in other plant groups (Hoppe‐Dominik, 1988). Indeed, undigested Poaceae and Cyperaceae plants were detected in 40%–50% of the scat of leopards (Hart et al., 1996) and tigers (Tkachenko, 2012), similar to that in the scat of puma and jaguarundi (Rocha‐Mendes et al., 2010). Therefore, these plant species may be consumed not for medicinal secondary compounds but for physical traits such as hairs on their surface (Hoppe‐Dominik, 1988).

Snow leopards and leopards have been reported to eat *Myricaria* shrubs in addition to grasses (Jumabay‐Uulu et al., 2014; Lovari et al., 2013; Wegge et al., 2012). Tamaricaceae plants (the family that includes *Myricaria*) have been detected in 4.1%–16.9% of scat and constituted the bulk of hairballs (Lovari et al., 2013), although it is uncertain that hairballs were caused by plant intake. These *Myricaria* plants have anti‐inflammatory properties and have been used as traditional medicines (Chernonosov et al., 2017; Liu et al., 2009). Cold and dry climates restrict the transmission and growth of parasites (Morris, 2002), whereas low temperature increases the probability of infection in the alpine hare (Schai‐Braun et al., 2019). As such, snow leopards, which had the highest frequency of plant occurrence among *Panthera* species, may utilize medicinal compounds derived from plants against parasites. Further, the relatively high frequency of plant occurrence reported in snow leopards that live in alpine environments where plants are scarce, together with no correlation with NDVI, support the possibility that plant consumption has some advantage for carnivores.

## APPENDIX 2

A list of parameters in the models.

**TABLE A1:** Details of parameters in the models

Parameters	Category	Size	Description
*N_*all	Integer	1	The total number of records
*N_*sp	Integer	1	The total number of species
*y*	Vector	*N*_all	The number of samples contained plant of each record
*ψ*	Numeric	1	The probability of reporting the frequency of plant occurrence
*N*	Vector	*N*_all	Sample size of each record
*p*	Vector	*N*_all	The frequency of plant occurrence of each record
*α*	Vector	*N*_sp	Species‐specific intercept
*τ*	Vector	*N*_all	Random effect of records
*θ*	Vector	*N*_sp	Hyperparameter for *τ*
*β*	Vector	*s*	Coefficient of environmental factors
*X_*env	Matrix	*s* × *N*_all	Environmental factor
*s*	Integer	1	The number of environmental factors
*I*	Numeric	1	Species‐independent intercept
*ε*	Vector	*N*_sp	Coefficient of nonenvironmental factors
*X_*sp	Matrix	*t* × *N*_sp	Nonenvironmental factor
*t*	Integer	1	The number of nonenvironmental factors
*φ*	Vector	*N*_sp	Random effect of species
*ω*	Vector	1	Hyperparameter for *φ*
