# Corrigendum

**DOI:** 10.1002/ece3.8148

**Published:** 2021-10-07

In the recent article by Yoshimura et al. (2021), the first and third sentences have been corrected in Section 2.2 as follows:

We included six environmental attributes: island, mean monthly precipitation, mean maximum daily temperature, mean minimum daily temperature, mean monthly normalized difference vegetation index (NDVI), and season (spring, summer, autumn, winter, dry, wet).

Precipitation and temperature represent climate parameters of the habitat of subject animals.

Figure 1 has also been updated as follows:

**FIGURE 1:**
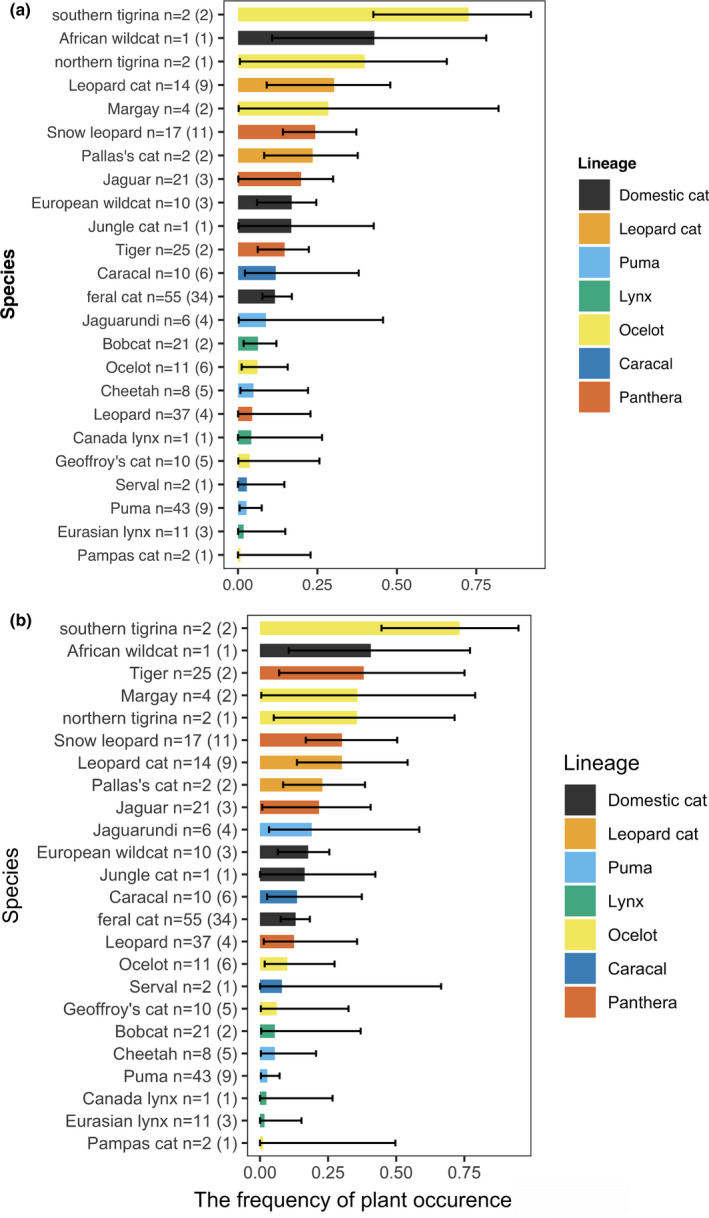
Estimated frequency of plant occurrence (a) maximum a posteriori estimate with the 89% highest density interval (HDI) and (b) expected a posteriori estimate with the 95% credible interval (CI) of each species using Model 1_3. The numbers next to the common names of species represent the numbers of records, and the numbers in the parentheses are the numbers of records showing the frequency of plant occurrence values

The authors apologize for the errors.

## References

[ece38148-bib-0001] Yoshimura, H. , Hirata, S. , & Kinoshita, K. (2021). Plant‐eating carnivores: Multispecies analysis on factors influencing the frequency of plant occurrence in obligate carnivores. Ecology and Evolution, 11, 10968–10983. 10.1002/ece3.7885 34429895 PMC8366844

